# Real-world outcomes of rivaroxaban treatment in elderly Japanese patients with nonvalvular atrial fibrillation

**DOI:** 10.1007/s00380-019-01487-x

**Published:** 2019-09-06

**Authors:** Takanari Kitazono, Takanori Ikeda, Satoshi Ogawa, Jyoji Nakagawara, Kazuo Minematsu, Susumu Miyamoto, Yuji Murakawa, Mary Cavaliere, Yasuhiro Hayashi, Yoko Kidani, Yutaka Okayama, Toshiyuki Sunaya, Shoichiro Sato, Satoshi Yamanaka

**Affiliations:** 1 Department of Medicine and Clinical Science, Graduate School of Medical Sciences, Kyushu University, 3-1-1 Maidashi, Higashi-ku, Fukuoka, 812-8582 Japan; 2 Department of Cardiovascular Medicine, Toho University Graduate School of Medicine, Tokyo, Japan; 3 International University of Health and Welfare Mita Hospital, Tokyo, Japan; 4 Osaka Namba Clinic, Osaka, Japan; 5 National Cerebral and Cardiovascular Center, Suita, Osaka Japan; 6 Iseikai Medical Corporation, Osaka, Japan; 7 Department of Neurosurgery, Kyoto University Graduate School of Medicine, Kyoto, Japan; 8 The 4th Department of Internal Medicine, Teikyo University School of Medicine, Mizonokuchi Hospital, Kawasaki, Japan; 9 Medical Affairs Thrombosis, Medical Affairs, Bayer Yakuhin, Ltd., Osaka, Japan; 10 Pharmacovigilance Monitoring and Medical Governance, Medical Affairs, Bayer Yakuhin, Ltd., Osaka, Japan; 11 Research and Development Japan/Data Sciences and Analytics/Statistics and Data Insights, Bayer Yakuhin, Ltd., Osaka, Japan

**Keywords:** Rivaroxaban, Atrial fibrillation, Anticoagulant, Elderly, Benefit–risk balance

## Abstract

Direct oral anticoagulants (DOACs), such as rivaroxaban, reduce the risk of stroke and systemic embolism in patients with nonvalvular atrial fibrillation (NVAF). However, it is still unclear whether the stroke reduction benefit outweighs the bleeding risk in elderly Japanese patients with NVAF. The Xarelto Post-Authorization Safety and Effectiveness Study in Japanese Patients with Atrial Fibrillation (XAPASS) was a real-world, prospective observational, post-marketing surveillance study on the safety and effectiveness of rivaroxaban in Japanese clinical practice. This sub-analysis evaluated the clinical outcomes of elderly patients aged ≥ 75 years. At the 1-year follow-up, there were 4,685 (48.91%) and 4,893 (51.09%) patients aged ≥ 75 and < 75 years, respectively. Safety and effectiveness outcomes were compared between patients aged ≥ 75 years and those aged < 75 years, and among 3 elderly sub-populations (age ranges: 75–79, 80–84, and ≥ 85 years). Patients aged ≥ 75 years had higher rates of major bleeding [2.22 vs. 1.35 events per 100 patient-years, hazard ratio (HR) 1.63, 95% confidence interval (CI) 1.17–2.28] and composite of stroke (ischemic or hemorrhagic)/non-central nervous system (non-CNS) systemic embolism (SE)/myocardial infarction (MI) (2.41 vs. 1.21 events per 100 patient-years, HR 1.97, 95% CI 1.40–2.77) compared to patients aged < 75 years. Intracranial hemorrhage rates were < 1 event per 100 patient-years in both groups (0.85 vs. 0.59 events per 100 patient-years, HR 1.43, 95% CI 0.85–2.40). Kaplan–Meier curves of major bleeding and stroke/non-CNS SE/MI showed that no significant differences of cumulative event rates were identified among the 3 elderly sub-populations. Stepwise Cox regression analyses revealed that creatinine clearance (CrCl) (<50 mL/min), hepatic impairment, and hypertension were specific predictors for major bleeding and no specific predictors were found for stroke/non-CNS SE/MI in patients aged ≥ 75 years. In conclusion, safety and effectiveness event rates were higher in patients aged ≥ 75 years compared with those aged < 75 years, yet, no distinct differences were observed among the 3 elderly sub-populations.

**Electronic supplementary material:**

The online version of this article (10.1007/s00380-019-01487-x) contains supplementary material, which is available to authorized users.

## Introduction

Atrial fibrillation (AF) is a growing epidemic and an important public health problem as the world population ages. In particular, AF patients in an elderly population are of considerable clinical interest, since they are at a high risk for not only ischemic stroke [1], but also bleeding that derives from multiple complicating factors, including comorbidities, polypharmacotherapy, cognitive deficits, and falling [2, 3]. Due to those complexities, practicing physicians often must perform a delicate balance and proper decision making. In addition, the decision making for such an elderly patient population is a challenge due to the limited availability of clinical and real-world data.

Rivaroxaban is a direct factor Xa inhibitor; the safety and efficacy of rivaroxaban were examined and compared with warfarin as a standard treatment. In the ROCKET AF study, which included 14,264 patients with nonvalvular atrial fibrillation (NVAF) worldwide, rivaroxaban demonstrated non-inferiority to warfarin for the prevention of stroke or systemic embolism [4]. In a similar study, the J-ROCKET AF study, focusing on Japanese patients and Japan-specific dosage, rivaroxaban demonstrated non-inferiority to warfarin for the principal safety outcome of major and non-major clinically relevant bleeding in patients with NVAF [5]. As the patient backgrounds, including age, may be less diverse in clinical trial settings, real-world studies will become more and more important in this regard [6].

Japan is a front runner of super-aged societies [7] with the highest average life expectancy in the world at 84 years (87 years for women and 80 years for men) [8, 9]. The prevalence of AF has been increasing in Japan as the population ages, as in Western countries, and the rate is projected to be 0.91% in 2030 and 1.09% in 2050 [10]. Therefore, there is a growing interest in the real-world evidence of direct oral anticoagulant treatment for elderly patients with NVAF.

As a real-world, post-authorization, prospective, single-arm, non-interventional cohort study, we performed the XAPASS that evaluated the safety and effectiveness of rivaroxaban in Japanese patients with NVAF [11]. The results revealed low incidence rates of bleeding and thromboembolic events, suggesting that rivaroxaban is safe and effective for stroke prevention in daily clinical practice [12]. The current study is a sub-analysis of the XAPASS focusing on elderly patients aged ≥ 75 years. The safety and effectiveness outcomes and possible predictive factors of major bleeding and thromboembolic events were evaluated. The sub-analysis provides additional information that may help facilitate treatment decisions for elderly patients aged ≥ 75 years in Japan.

## Materials and methods

### Study design

The XAPASS (Clinicaltrials.gov: NCT01582737) was undertaken as a real-world, prospective, open-label, single-arm, observational, post-authorization cohort study conducted in Japan. The study design was described previously [11]. Briefly, the standard observation period for each patient is 2 years; data are collected at 6 months, 1 year, and 2 years after the initiation of rivaroxaban treatment. After the completion of the standard observation period, follow-up investigations are being conducted for a maximum for 5 years. The study was approved by the Ministry of Health, Labour, and Welfare in Japan and was carried out in accordance with the standards for Good Post-marketing Study Practice (GPSP) provided by this ministry. Individual consent and institutional approval of ethical standards in accordance with the Declaration of Helsinki are not necessary in activities and research for the safety surveillance, such as signal detection and prospective cohort studies.

### Patients

A total of 11,308 Japanese patients with NVAF were enrolled in the XAPASS between April 2012 and June 2014. The current sub-analysis included 9,578 patients who had completed a 1-year follow-up as of September 2017.

### Treatment

Patients received oral rivaroxaban at a dosage of either 15 mg once daily (od) or 10 mg od, prescribed at the discretion of the treating physicians. These dosages are approved in Japan for patients with CrCl ≥ 50 and < 50 mL/min, respectively.

### Risk scores

The following scores, as determined at baseline to assess stroke risk, were compared in patients aged ≥ 75 years and patients aged < 75 years: CHADS_2_ (congestive heart failure, hypertension, age, diabetes mellitus, stroke) [13] and CHA_2_DS_2_-VASc (congestive heart failure, hypertension, age ≥ 75 years, diabetes mellitus, stroke, vascular disease, age 65–74 years, sex category) [14]. A modified HAS-BLED score, also determined at baseline to assess bleeding risk, included the following factors: hypertension, abnormal liver/renal function, stroke history, bleeding predisposition, elderly, and drug/alcohol use. The labile international normalized ratio was excluded from the score [12, 15].

### Study outcomes

The primary safety outcome was any bleeding. Major bleeding and intracranial haemorrhage (ICH) were recorded as the components. Major bleeding was defined according to the International Society of Thrombosis and Haemostasis criteria [16], whereas non-major bleeding was defined as any bleeding that did not meet these criteria. The primary effectiveness outcome was a composite of stroke (hemorrhagic or ischemic), non-central nervous system (non-CNS) SE, and myocardial infarction (MI). All the outcomes were defined previously [11, 12]. Stroke and ischemic stroke were recorded as individual outcomes. Transient ischemic attack (TIA) was not included in the stroke endpoint.

### Statistical analysis

Survival curves were estimated by the Kaplan–Meier method. Cox regression analysis was performed to estimate hazard ratios of outcomes, which were compared between patients aged ≥ 75 and < 75 years, and among the 3 elderly sub-populations (age ranges: 75–79, 80–84, and ≥ 85 years). *P* values were calculated using the log-rank test. A competing risk analysis was performed using the Fine and Gray’s proportional subhazards model [17] to compare the cumulative incidence function of major bleeding and stroke/non-CNS SE/MI events among the 3 elderly sub-populations with ‘death before event occurrence’ as the competing risk. Predictive factors for major bleeding and stroke/non-CNS SE/MI events in patients aged ≥ 75 and < 75 years were estimated by multivariable and stepwise analyses using the Cox proportional hazards model with a significance level of 5%. The following variables at the study enrollment were included: female gender, body weight, CrCl, initial dose, hypertension, diabetes mellitus, congestive heart failure, prior ischemic stroke/TIA, vascular disease, hepatic dysfunction, and oral antiplatelet use. *P* < 0.05 was considered significant for the analysis. All statistical analyses were performed using SAS version 9.2 or higher (SAS Institute Inc., Cary, NC).

## Results

### Patients

Baseline characteristics are shown in Table 1. Of the 9,578 patients who completed the 1-year follow-up, 4,685 (48.91%) patients were aged ≥ 75 years. These patients were more likely to be female than patients aged < 75 years (47.51% vs. 29.25%) and had a lower body weight (mean: 56.80 ± 11.83 kg, vs. 65.75 ± 12.73 kg). Patients aged ≥ 75 years had higher rates of CrCl < 50 mL/min (41.86% vs. 6.58%), and higher rates of the following comorbidities: hypertension (78.61% vs. 71.45%), prior ischemic stroke/TIA (27.85% vs. 17.33%), and congestive heart failure (29.28% vs. 20.56%). In accordance with these higher comorbidity rates, patients aged ≥ 75 years had higher CHADS_2_, CHA_2_DS_2_-VASc, and modified HAS-BLED scores (mean: 2.9 ± 1.2 vs. 1.5 ± 1.1, 4.4 ± 1.3 vs. 2.5 ± 1.4, and 1.9 ± 0.9 vs. 1.2 ± 0.9, respectively).

**Table 1:** Baseline patient characteristics

	< 75 years	≥ 75 years
Characteristic	(*N* = 4,893)	(*N* = 4,685)
Age, years	65.7 ± 7.4	80.9 ± 4.6
Female sex	1,431 (29.25)	2,226 (47.51)
Body weight, kg	65.75 ± 12.73	56.80 ± 11.83
Body weight, kg [*n* (%)]		
≤ 50	473 (9.67)	1392 (29.71)
> 50	4,076 (83.30)	2,952 (63.01)
Unknown	344 (7.03)	341 (7.28)
BMI, kg/m^2^	24.51 ± 3.98	23.27 ± 4.07
CrCl, mL/min	81.4 ± 31.3	53.5 ± 18.6
CrCl, mL/min [*n* (%)]		
< 50	322 (6.58)	1,961 (41.86)
≥ 50	4,173 (85.29)	2,348 (50.12)
Unknown	398 (8.13)	376 (8.03)
CHADS_2_ score, mean ± SD	1.5 ± 1.1	2.9 ± 1.2
Score, *n* (%)		
0	842 (17.21)	0
1	1,886 (38.54)	447 (9.54)
2	1,216 (24.85)	1,687 (36.01)
3	677 (13.84)	1,199 (25.59)
4	231 (4.72)	875 (18.68)
5	41 (0.84)	387 (8.26)
6	0	90 (1.92)
25th percentile	1	2
Median	1	3
75th percentile	2	4
CHA_2_DS_2_-VASc score	2.5 ± 1.4	4.4 ± 1.3
Score		
0	258 (5.27)	0
1	908 (18.56)	0
2	1,405 (28.71)	228 (4.87)
3	1,238 (25.30)	1,008 (21.52)
4	691 (14.12)	1,467 (31.31)
5	302 (6.17)	1,077 (22.99)
6	80 (1.63)	620 (13.23)
7	11 (0.22)	239 (5.10)
8	0	44 (0.94)
9	0	2 (0.04)
25th percentile	2	3
Median	2	4
75th percentile	3	5
Modified HAS-BLED score*	1.2 ± 0.9	1.9 ± 0.9
Score		
0	1,211 (24.75)	0
1	2,128 (43.49)	1,813 (38.70)
2	1,148 (23.46)	1,860 (39.70)
3	333 (6.81)	818 (17.46)
4	65 (1.33)	173 (3.69)
5	6 (0.12)	20 (0.43)
6	0	1 (0.02)
7	0	0
8	0	0
25th percentile	1	1
Median	1	2
75th percentile	2	2
Baseline comorbidities		
Hypertension	3,496 (71.45)	3,683 (78.61)
Diabetes mellitus	1,182 (24.16)	957 (20.43)
Prior ischemic stroke/TIA	848 (17.33)	1,305 (27.85)
Congestive heart failure	1,006 (20.56)	1,372 (29.28)
Hepatic dysfunction	360 (7.36)	228 (4.87)
Type of AF		
Paroxysmal	1,728 (35.32)	1,493 (31.87)
Persistent	1,724 (35.23)	1,702 (36.33)
Permanent	1,192 (24.36)	1,151 (24.57)
Other	10 (0.20)	12 (0.26)
Unknown	239 (4.88)	327 (6.98)

Data are presented as *n* (%) or mean ± standard deviation

*AF* atrial fibrillation, *BMI* body mass index, *CrCl* creatinine clearance, *INR* international normalized ratio, *TIA* transient ischemic attack

^*^Maximum score is 8 because of the exclusion of the factor "labile INR" from the HAS-BLED score

### Safety outcomes

**Fig. 1:**
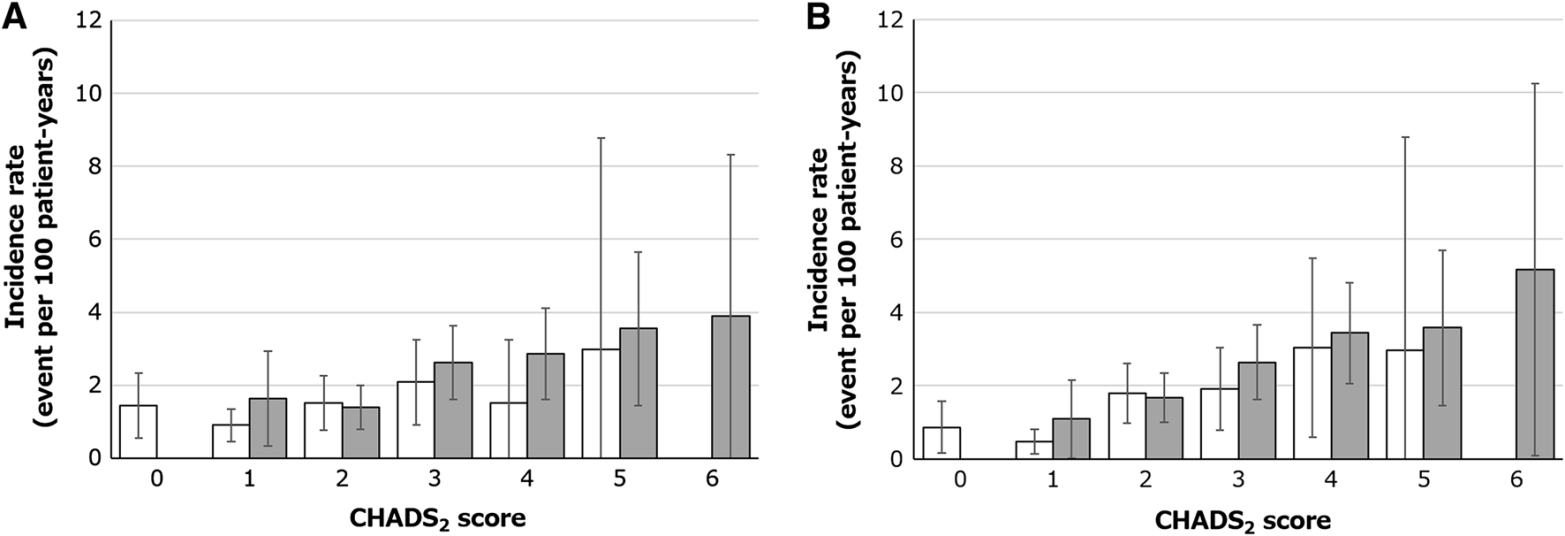
Incidence rates of **a** major bleeding and **b** stroke/non-CNS SE/MI stratified by baseline CHADS_2_ score. Gray and white bar indicate the rates for patients aged ≥ 75 and < 75 years, respectively. Error bars indicate the corresponding 95% confidence interval

**Fig. 2:**
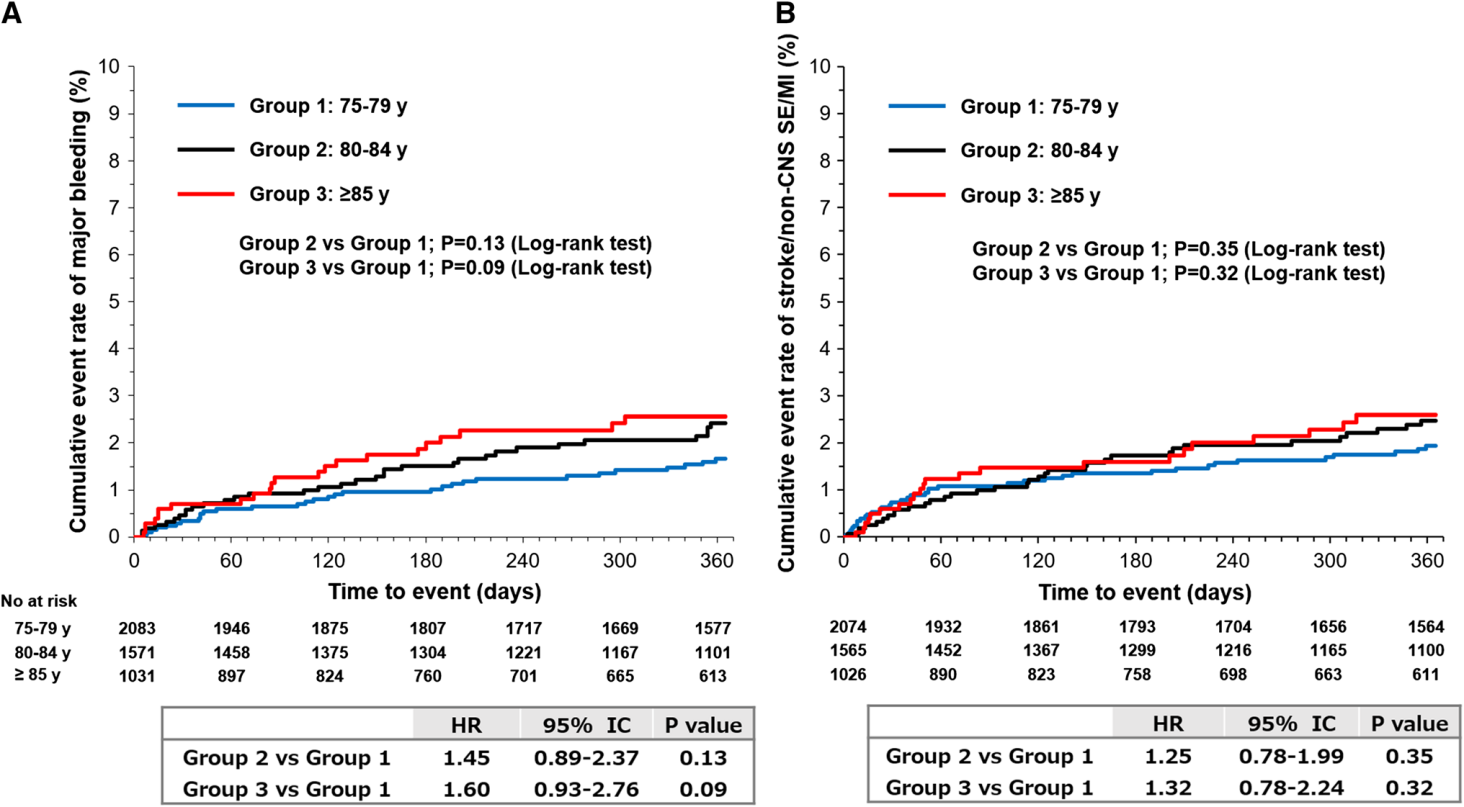
Kaplan–Meier curves for the cumulative event rate of **a** major bleeding and **b** stroke/non-CNS SE/MI among the three elderly patient sub-groups (groups 1–3: ages 75–79, 80–84, and ≥ 85 years, respectively) with NVAF

Patients aged ≥ 75 years had higher rates of any bleeding (8.58 vs. 6.74 events per 100 patient-years, HR 1.26, 95% CI 1.07–1.48), major bleeding (2.22 vs. 1.35 events per 100 patient-years, HR 1.63, 95% CI 1.17–2.28), and fatal bleeding (0.28 vs. 0.09 events per 100 patient-years, HR 2.99, 95% CI 0.95–9.38) compared to patients aged < 75 years old (Table 2). ICH rates were < 1% in both age groups (0.85 vs. 0.59 events per 100 patient-years, HR 1.43, 95% CI 0.85–2.40). In both patients aged ≥ 75 and < 75 years stratified by CHADS_2_ score, incidence rates of major bleeding trended higher with higher CHADS_2_ score (Fig. 1a). Cox regression analysis showed that there was no significant increased rates in major bleeding (HR 1.45, 95% CI 0.89–2.37 for 80–84 years vs. 75–79 years and HR 1.60, 95% CI 0.93–2.76 for ≥ 85 years vs. 75–79 years) (Fig. 2a). We note that incidence rate of death was 1.71, 4.79, and 8.47 events per 100 patient-years for patients aged 75–79, 80–84, and ≥ 85 years, respectively (Supplementary Fig. 1). A competing risk analysis showed that no significant difference in the incidence rates of major bleeding among the three elderly age groups even with adjustment for mortality was observed (Supplementary Table 1).

**Table 2:** Safety and effectiveness outcomes

	< 75 years		≥ 75 years		HR (95% CI), ≥ 75 years vs. < 75 years	*P* value
	Incidence proportion, *n* (%)	Incidence rate, event per 100 patient-years (95% CI)	Incidence proportion, *n* (%)	Incidence rate, event per 100 patient-years (95% CI)		
Safety outcome	(*N* = 4,893)		(*N* = 4,685)			
Any bleeding	278 (5.68)	6.74 (5.95–7.54)	324 (6.92)	8.58 (7.64–9.51)	1.26 (1.07–1.48)	0.005
Major bleeding	57 (1.16)	1.35 (1.00–1.70)	86 (1.84)	2.22 (1.75–2.68)	1.63 (1.17–2.28)	0.004
Fatal bleeding	4 (0.08)	0.09 (0.00–0.19)	11 (0.23)	0.28 (0.12–0.45)	2.99 (0.95–9.38)	0.049
Critical organ bleeding	27 (0.55)	0.64 (0.40–0.88)	38 (0.81)	0.98 (0.67–1.29)	1.52 (0.93–2.49)	0.093
Intracranial hemorrhage	25 (0.51)	0.59 (0.36–0.82)	33 (0.70)	0.85 (0.56–1.34)	1.43 (0.85–2.40)	0.179
Hemoglobin decrease ≥ 2 g/dl	20 (0.41)	0.47 (0.27–0.68)	30 (0.64)	0.77 (0.49–1.05)	1.62 (0.92–2.86)	0.090
Transfusion of ≥ 2 units of packed RBC or whole blood	6 (0.12)	0.14 (0.03–0.25)	13 (0.28)	0.33 (0.15–0.51)	2.33 (0.89–6.13)	0.077

Effectiveness outcome	(*N* = 4,878)		(*N* = 4,665)			
Stroke/non-CNS SE/MI	51 (1.05)	1.21 (0.88–1.54)	93 (1.99)	2.41 (1.92–2.89)	1.97 (1.40–2.77)	< 0.001
Stroke	48 (0.98)	1.14 (0.82–1.46)	80 (1.71)	2.07 (1.62–2.53)	1.80 (1.26–2.57)	0.001
Ischemic stroke	31 (0.64)	0.73 (0.48–0.99)	60 (1.29)	1.55 (1.16–1.95)	2.09 (1.35–3.22)	< 0.001

### Effectiveness outcomes

Patients aged ≥ 75 years had higher rates of the composite endpoint stroke/non-CNS SE/MI compared with those aged < 75 years (2.41 vs. 1.21 events per 100 patient-years, HR 1.97, 95% CI 1.40–2.77; Table 2). They also had higher rates of the individual endpoints of stroke (2.07 vs. 1.14 events per 100 patient-years, HR 1.80, 95% CI 1.26–2.57) and ischemic stroke (1.55 vs. 0.73 events per 100 patient-years, HR 2.09, 95% CI 1.35–3.22). In both patients aged ≥ 75 and < 75 years stratified by CHADS_2_ score, there was a similar tendency for incidence rates of stroke/non-CNS SE/MI to increase as the rates of major bleeding increased (Fig. 1b). Cox regression analysis showed that there was no significant increased rates in stroke/non-CNS SE/MI (HR 1.25, 95% CI 0.78–1.99 for 80–84 years vs. 75–79 years and HR 1.32, 95% CI 0.78–2.24 for ≥ 85 years vs. 75–79 years) (Fig. 2b). A competing risk analysis showed similar results (Supplementary Table 1).

### Predictive factors for major bleeding and stroke/non-CNS SE/MI events

In multivariable analyses, CrCl < 50 mL/min (HR 1.84, 95% CI 1.09–3.12; *P* = 0.023), hypertension (HR 2.12, 95% CI 1.01–4.43; *P* = 0.046), hepatic dysfunction (HR 2.32, 95% CI 1.11–4.85; *P* = 0.025), and oral antiplatelet use (HR 2.54, 95% CI 1.25–5.18; *P* = 0.010) were found to be associated with major bleeding events in patients aged ≥ 75 years (Table 3), while hypertension (HR 0.55, 95% CI 0.31–0.97; *P* = 0.038), diabetes mellitus (HR 1.92, 95% CI 1.10–3.37; *P* = 0.023), and oral antiplatelet use (HR 2.98, 95% CI 1.26–7.05; *P* = 0.013) were identified in patients aged < 75 years (Supplementary Table 2). Prior ischemic stroke/TIA (HR 1.78, 95% CI 1.16–2.74; *P* = 0.009) was associated with stroke/non-CNS SE/MI events in patients aged ≥ 75 years (Table 3), while body weight (HR 2.73, 95% CI 1.23–6.04; *P* = 0.014), prior ischemic stroke/TIA (HR 2.27, 95% CI 1.24–4.13; *P* = 0.008), and oral antiplatelet use (HR 4.47, 95% CI 2.07–9.65; *P* < 0.001) were identified in patients aged < 75 years (Supplementary Table 2). Importantly, stepwise regression analysis suggested that CrCl < 50 mL/min, hepatic dysfunction, and hypertension were specific predictive factors of major bleeding and no factors were specifically predictive of stroke/non-CNS SE/MI for patients aged ≥ 75 years (Supplementary Table 3).

**Table 3:** Cox regression analysis for major bleeding and stroke/non-CNS SE/MI in patients aged ≥ 75 years

	Major Bleeding				Stroke/non-CNS SE/MI			
	Univariable analysis		Multivariable analysis		Univariable analysis		Multivariable analysis	
Variables	HR (95% CI)	*P* value	HR (95% CI)	*P* value	HR (95% CI)	*P* value	HR (95% CI)	*P* value
Female gender								
Yes/no	1.36 (0.89–2.08)	0.156	1.34 (0.82–2.19)	0.249	0.81 (0.54–1.22)	0.310	0.88 (0.55–1.41)	0.597
Body weight, kg								
> 50 vs. ≤ 50	1.39 (0.88–2.19)	0.153	1.13 (0.66–1.93)	0.669	0.91 (0.58–1.44)	0.697	0.84 (0.49–1.44)	0.517
CrCl, mL/min								
≥ 50 vs. < 50	1.76 (1.13–2.76)	0.013^*^	1.84 (1.09–3.12)	0.023^*^	1.31 (0.86–1.99)	0.205	1.42 (0.87–2.31)	0.158
Initial dose								
10 mg vs. 15 mg	1.12 (0.69–1.82)	0.643	0.71 (0.41–1.23)	0.225	0.94 (0.60–1.46)	0.771	0.83 (0.50–1.39)	0.481
Hypertension								
Yes/no	1.77 (0.94–3.33)	0.077	2.12 (1.01–4.43)	0.046^*^	1.47 (0.83–2.60)	0.183	1.45 (0.80–2.64)	0.218
Diabetes mellitus								
Yes/no	1.39 (0.86–2.24)	0.179	1.38 (0.84–2.27)	0.208	0.84 (0.50–1.43)	0.530	0.78 (0.46–1.34)	0.374
CHF								
Yes/no	1.14 (0.72–1.80)	0.569	1.00 (0.61–1.62)	0.992	1.19 (0.77–1.83)	0.441	1.28 (0.81–2.00)	0.291
Prior ischemic stroke/TIA								
Yes/no	1.46 (0.93–2.27)	0.097	1.38 (0.86–2.20)	0.177	2.04 (1.36–3.08)	< 0.001^*^	1.78 (1.16–2.74)	0.009^*^
Vascular disease								
Yes/no	0.91 (0.29–2.86)	0.865	0.74 (0.23–2.39)	0.610	1.43 (0.58–3.52)	0.435	0.95 (0.34–2.65)	0.928
Hepatic dysfunction								
yes/no	2.01 (0.97–4.16)	0.060	2.32 (1.11–4.85)	0.025^*^	1.11 (0.45–2.73)	0.821	1.18 (0.48–2.92)	0.724
Oral antiplatelet use								
Yes/no	2.87 (1.48–5.55)	0.002^*^	2.54 (1.25–5.18)	0.010^*^	2.07 (1.00–4.28)	0.049^*^	1.84 (0.88–3.86)	0.108

*CHF* congestive heart failure, *CI* confidence interval, *CrCl* creatinine clearance, *HR* hazard ratio, *TIA* transient ischemic attack

*P < 0.05

## Discussion

This sub-analysis was aimed at evaluating the safety and effectiveness of rivaroxaban treatment for stroke prevention in NVAF patients aged ≥ 75 years. These patients presented higher rates of both major bleeding and the composite endpoint of stroke/non-CNS SE/MI compared to patients aged < 75 years. Our results were in agreement with those in the studies of Hori et al. [18] and Lip et al. [1], which showed increased rates of major bleeding and stroke/thromboembolism rates with advancing age in patients with NVAF. One possible explanation for our results was that impaired kidney function (CrCl < 50 mL/min) occurred more frequently in patients aged ≥ 75 years than in those aged < 75 years (41.86% vs. 6.58% of patients, respectively). Chronic kidney disease is known to be associated with increased risks of bleeding and stroke in NVAF patients under anticoagulation therapy [19]. Another possible reason was the higher prevalence of cardiovascular conditions with consequent higher risk scores, such as CHADS_2_, CHA_2_DS_2_-VASc, and modified HAS-BLED, in patients aged ≥ 75 years. Of note, there was no significant difference in the ICH rate between the two age groups treated with rivaroxaban. In both groups, the ICH rate was < 1 event per 100 patient-years, which was consistent with the pivotal studies of ROCKET AF [4] and J-ROCKET AF [5]. A possible explanation for the similar ICH rate between the two age groups in our sub-analysis could be partially explained by physicians’ careful management of comorbidities and concomitant medications. We speculate that modifiable bleeding risk factors, such as blood pressure, invasive procedures, and concomitant medications, were appropriately controlled especially in patients aged ≥ 75 years. The Shikoku Rivaroxaban Registry Trial in Japanese real-world settings reported similarly that there was no significant difference in the ICH rate between extremely elderly patients (≥ 80 years) and control patients (< 80 years) [20]. Further study will be needed to examine the relationship between age and ICH rate in patients taking DOACs.

An interesting finding was that there was no significant difference in major bleeding or stroke/non-CNS SE/MI events among the 3 elderly sub-populations (age ranges: 75–79, 80–84, and ≥ 85 years). Since the target population was very old and the mortality rate of this population was higher than the thromboembolic and major bleeding rates particularly for patients over 80 years old (Supplementary Fig. 1), we performed the competing risk analysis to evaluate the impact of death. The comparison of hazard ratios obtained with Cox regression and the competing risk analysis suggested that the impact of mortality on major bleeding and stroke/non-CNS SE/MI outcomes was negligible (Supplementary Table 1). Emerging evidences indicate that ignoring the competing death risk can lead to biased estimates of stroke risks especially in elderly AF patients [21, 22]; however, this was not the case in this analysis. One possible reason could be a shorter follow-up period (1 year), which might have limited the effect of death rate. Follow-up data collected over a longer period analyzed with the competing risk method will be required to access the impact of the observation period. Despite the limitation mentioned above, our findings may be attributed in part to the “healthy survivor effect” [23], meaning that one would be sufficiently healthy to be alive. We presume the elderly sub-populations were healthy enough and, as a result, their thromboprophylaxis outcomes did not differ significantly among the sub-populations. Indeed, no higher prevalence of comorbidities was found among the sub-populations, even though the mean CrCl values were sharply decreased with increasing age (Supplementary Table 4). Also, advancing chronological age is not always directly linked to poor health, which suggests that the chronological age number should not be the only factor used as a health index. Furthermore, careful risk–benefit assessment or management of patients by the physicians may give better results across all the elderly sub-populations. However, the results of the XAPASS study should be interpreted carefully, since bias may have been introduced into the outcome data, which is one of the limitations of post-marketing surveillance studies.

Currently, the elderly population continues to age and a longer life expectancy has become increasingly common in certain countries [24]. Japan has already entered into the era of a super-aged society. As an example, in the 1-year results of the XAPASS, the average age was 73.2 ± 9.8 [12], and our result showed that almost half of the 9,578 patients with NVAF were aged ≥ 75 years. The average age was similar to that found in various registries throughout Japan, including the EXPAND, SAKURA AF, RAFFINE, and Fushimi AF registries [25–28]. This impressive demographic aging is related to an undeniable social and economic burden, and a balanced approach in elderly medical care settings is imperative for the sustainability of the healthcare system [1]. Several lines of evidence indicate that DOACs are cost effective compared to warfarin, yet it is still controversial whether lines of evidence apply to elderly patients (≥ 75 years) in real-world settings since they have increased bleeding risks compared to younger elderly patients (65–74 years) [29]. Therefore, decisions on appropriate AF treatment should ultimately be individualized and should balance treatment-related benefits, risks, cost, and patient preferences.

The use of anticoagulation requires risk–benefit assessment, especially for elderly patients with AF. First, even though bleeding concern is a serious issue, there are several findings which support the use of oral anticoagulants (OACs) in elderly for the prevention of thromboembolic complications of AF, such as, the recently published PREFER in AF Registry conducted in European countries [30]. The incidence rate of thromboembolic events, which includes stroke/TIA/systemic embolism, was higher in the very elderly (≥ 85 years) than in younger (< 85 years) AF patients, and the use of OACs resulted in a greater absolute reduction in the incidence rate in elderly (≥ 85 years) than in younger (< 85 years) AF patients (2% vs. 0.5%). In elderly patients (≥ 85 years), the risk of major bleeding was higher than in younger (< 85 years) patients, but similar in patients on OACs and in those on antiplatelet therapy or without antithrombotic treatment. Second, Chao TF et al. showed that very elderly patients with AF (≥ 90 years) still benefit from DOACs treatment [31]. DOACs may be considered for stroke prevention in this extreme aged population, given the significant stroke risk reduction and the positive net clinical benefit. Also, DOACs were associated with a lower risk of ICH compared with warfarin. Third, in a sub-analysis of the ROCKET AF trial, rivaroxaban was found to have a higher net clinical benefit than warfarin in elderly patients [32], although it remains to be determined whether this is true in real-world Japanese clinical practice. We assume advanced age continues to be an important barrier to anticoagulation treatment as in the past [33], where stroke risk was underestimated and bleeding, lack of adherence, and falls risk are overestimated; however, such a recognition may become antiquated.

Careful risk assessment at the start of OACs is crucial to prevent adverse events in high risk elderly patients. Importantly, minimizing any modifiable risk factors such as uncontrolled hypertension, concurrent antiplatelet agent use, non-steroidal anti-inflammatory drug use, and harmful alcohol consumption may decrease bleeding in such patients during protection against stroke using DOACs [34]. Additionally, close monitoring is necessary in elderly patients as their health condition could rapidly deteriorate. We investigated the predictive factors of major bleeding and stroke/non-CNS SE/MI events for the elderly patients. In our Cox regression analyses, CrCl < 50 mL/min and hepatic dysfunction were indicated as specific predictive factors for major bleeding in patients aged ≥ 75 years, while no specific predictive factor of stroke/non-CNS SE/MI was identified. Even though renal dysfunction and hepatic dysfunction are not necessarily medically manageable factors, this information may help physicians manage bleeding more effectively in such patients. We included hypertension as a specific predictive factor of major bleeding for patients aged ≥ 75 years, as it was negatively associated with major bleeding events in patients aged < 75 years. On the other hand, prior ischemic stroke/TIA in stroke/non-CNS SE/MI and oral antiplatelet use in major bleeding were identified for both patients aged ≥ 75 and < 75 years. A similar tendency was observed in a sub-analysis of the EXPAND study, which investigated risk factors for stroke/systemic embolism and major bleeding in Japanese NVAF patients receiving rivaroxaban [35].

There are some limitations of this sub-analysis. First, the XAPASS is a single-arm study. It is impossible to directly compare the outcomes of rivaroxaban treatment with those of other treatments such as warfarin and other DOACs, as previously described [12]. Second, the sub-analysis was an only 1-year follow-up, limiting the ability to assess for late clinical events. Third, the loss of patients to follow-up might have also led to an underestimation of the event rates. Fourth, there might be some potential selection bias by the physicians.

In conclusion, a specific caution should be implemented when it comes to the treatment of elderly patients with NVAF. However, we understand with the aging of the world population, elderly patients should not be left untreated. The previous concept of not offering an available and indicated therapy simply because of “old age” may need to be revisited. Individualized analysis of the medical history and meticulous risk assessment should be considered in the decision making to decrease risk and increase the quality of life benefits for this unique population.

## Electronic supplementary material

Below is the link to the electronic supplementary material.

Supplementary file1 (PDF 927 kb)
