# Supplementary material for: Real-world outcomes of rivaroxaban treatment in elderly Japanese patients with nonvalvular atrial fibrillation
Source: Heart Vessels. 2019 Sep 6;35(3):399–408. doi: 10.1007/s00380-019-01487-x (PMC7026217; doi:10.1007/s00380-019-01487-x)
Supplement: Supplementary file 1 — Supplementary file1 (PDF 927 kb) [file 380_2019_1487_MOESM1_ESM.pdf]

Supplementary Materials

Title: Real-world outcomes of rivaroxaban treatment in elderly Japanese patients with nonvalvular atrial fibrillation

Journal: Heart and Vessels

Authors: Takanari Kitazono[1], Takanori Ikeda[2], Satoshi Ogawa[3], Jyoji Nakagawara[4,5], Kazuo Minematsu[5,6], Susumu Miyamoto[7], Yuji Murakawa[8], Mary Cavaliere[9], Yasuhiro Hayashi[9], Yoko Kidani[9], Yutaka Okayama[10], Toshiyuki Sunaya[11], Shoichiro Sato[10] and Satoshi Yamanaka[9]

Affiliations:

1. Department of Medicine and Clinical Science, Graduate School of Medical Sciences, Kyushu University, Fukuoka, Japan
2. Department of Cardiovascular Medicine, Toho University Graduate School of Medicine, Tokyo, Japan
3. International University of Health & Welfare Mita Hospital, Tokyo, Japan
4. Osaka Namba Clinic, Osaka, Japan
5. National Cerebral and Cardiovascular Center, Suita, Osaka, Japan
6. Iseikai Medical Corporation, Osaka, Japan
7. Department of Neurosurgery, Kyoto University Graduate School of Medicine, Kyoto, Japan
8. The 4th Department of Internal Medicine, Teikyo University School of Medicine, Mizonokuchi Hospital, Kawasaki, Japan
9. Medical Affairs Thrombosis, Medical Affairs, Bayer Yakuhin, Ltd., Osaka, Japan

10. Pharmacovigilance Monitoring & Medical Governance, Medical Affairs, Bayer Yakuhin, Ltd., Osaka, Japan
11. Research & Development Japan/ Data Sciences & Analytics/ Statistics & Data Insights, Bayer Yakuhin, Ltd., Osaka, Japan

Corresponding author:

Takanari Kitazono, MD, PhD

Address: 3- 1- 1 Maidashi, Higashi-ku, Fukuoka 812-8582, Japan

Telephone: +81 92-642-5250

Fax: +81 92-642-5271

E-mail address: kitazono@intmed2.med.kyushu-u.ac.jp

Supplementary Fig.1. Kaplan Meier curves for the cumulative event rates for all-cause mortality in the three elderly patient sub-groups (Group 1-3: ages 75-79, 80-84, and ≥85 years, respectively) with NVAF

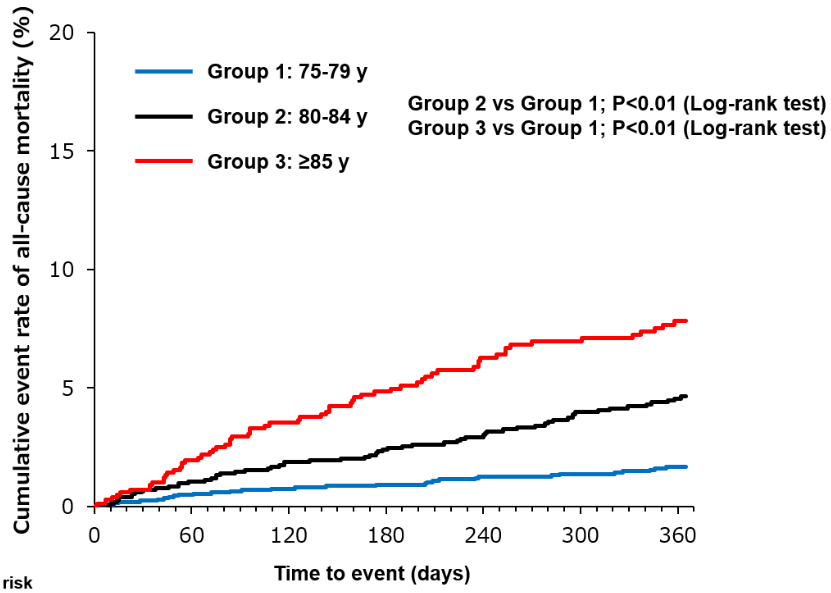

No at risk							
75-79 y	2083	1954	1883	1816	1727	1680	1589
80-84 y	1571	1465	1383	1316	1232	1178	1114
≥ 85 y	1031	899	830	766	706	670	618

	HR	95% IC	P value
Group 2 vs Group 1	2.79	1.81-4.28	<0.01
Group 3 vs Group 1	4.88	3.18-7.48	<0.01

	Incidence rate, event per 100 patient-years
Group 1	1.71
Group 2	4.79
Group 3	8.47

Supplementary Table 1. Comparison of hazard ratio between Cox regression and competing risk analysis in elderly NVAF patient sub-groups.

a) Major Bleeding

	Cox regression analysis[1)]		Competing risk analysis[2)]	
Age	HR (95% CI)	P value	HR (95% CI)	P value
75-79	1		1	
80-84	1.45 (0.89-2.37)	0.13	1.44 (0.88-2.35)	0.15
≥ 85	1.60 (0.93-2.76)	0.09	1.56 (0.90-2.69)	0.11

b) Stroke/non-CNS SE/MI

	Cox regression analysis[1)]		Competing risk analysis[2)]	
Age	HR (95% CI)	P value	HR (95% CI)	P value
75-79	1		1	
80-84	1.25 (0.78-1.99)	0.35	1.24 (0.78-1.97)	0.37
≥ 85	1.32 (0.78-2.24)	0.32	1.29 (0.76-2.19)	0.35

1) No competing risk was considered. HR (95% CI) and P value were the same as shown in Fig. 2.

2) Death before developing events was treated as a competing risk. Fine and Gray's proportional subhazards model was used.

Supplementary Table 2. Cox regression analysis for major bleeding and stroke/non-CNS SE/MI in patients aged <75 years

	**Major Bleeding**				**Stroke/non-CNS SE/MI**			
	**Univariable analysis**		**Multivariable analysis**		**Univariable analysis**		**Multivariable analysis**	
**Variables**	**HR (95% CI)**	***P* value**	**HR (95% CI)**	***P* value**	**HR (95% CI)**	***P* value**	**HR (95% CI)**	***P* value**
**Female gender**								
yes/ no	0.85 (0.47–1.53)	0.579	0.78 (0.40–1.52)	0.461	1.09 (0.60–1.96)	0.785	0.81 (0.41–1.61)	0.547
**Body weight, kg**								
>50 vs. ≤50	1.71 (0.84–3.50)	0.140	1.93 (0.83–4.54)	0.129	2.83 (1.48–5.42)	0.002*	2.73 (1.23–6.04)	0.014*
**CrCl, ml/min**								
≥50 vs. <50	1.93 (0.87–4.27)	0.104	1.72 (0.68–4.33)	0.252	3.40 (1.69–6.80)	0.001*	1.73 (0.77–3.89)	0.185
**Initial dose**								
10 mg vs. 15 mg	0.99 (0.55–1.76)	0.968	0.79 (0.41–1.53)	0.492	2.26 (1.30–3.92)	0.004*	1.77 (0.95–3.32)	0.074
**Hypertension**								
yes/ no	0.71 (0.41–1.21)	0.208	0.55 (0.31–0.97)	0.038*	1.01 (0.55–1.88)	0.965	0.93 (0.48–1.80)	0.830
**Diabetes mellitus**								
yes/ no	1.96 (1.15–3.34)	0.014*	1.92 (1.10–3.37)	0.023*	1.85 (1.05–3.26)	0.034*	1.74 (0.95–3.16)	0.071
**CHF**								
yes/ no	1.63 (0.93–2.88)	0.090	1.42 (0.78–2.58)	0.252	0.94 (0.47–1.87)	0.853	0.85 (0.42–1.75)	0.666
**Prior ischemic stroke/TIA**								
yes/ no	1.18 (0.61–2.27)	0.628	1.00 (0.50–2.01)	0.990	2.92 (1.66–5.15)	<0.001*	2.27 (1.24–4.13)	0.008*
**Vascular disease**								
yes/ no	3.12 (1.25–7.81)	0.015*	2.10 (0.77–5.74)	0.146	2.01 (0.63–6.47)	0.239	1.06 (0.31–3.62)	0.922
**Hepatic dysfunction**								
yes/ no	0.96 (0.35–2.66)	0.944	1.00 (0.36–2.78)	0.997	0.80 (0.25–2.56)	0.701	0.90 (0.28–2.91)	0.858
**Oral antiplatelet use**								
yes/ no	3.31 (1.50–7.31)	0.003*	2.98 (1.26–7.05)	0.013*	5.08 (2.47-10.4)	<0.001*	4.47 (2.07–9.65)	<0.001*

CHF, congestive heart failure; CI, confidence interval; CrCl, creatinine clearance; HR, hazard ratio; TIA, transient ischemic attack. *$P<0.05$

Supplementary Table 3. Predictive factors for major bleeding and stroke/non-CNS SE/MI

	Major Bleeding				Stroke/non-CNS SE/MI			
	< 75 years		≥ 75 years		< 75 years		≥ 75 years	
**Variables**	**HR (95% CI)**	***P* value**	**HR (95% CI)**	***P* value**	**HR (95% CI)**	***P* value**	**HR (95% CI)**	***P* value**
**Female gender**								
yes/ no								
**Body weight, kg**								
>50 vs. ≤50					2.52 (1.28–4.97)	0.008		
**CrCl, ml/min**								
≥50 vs. <50			1.80 (1.15–2.82)	0.010				
**Initial dose**								
10 mg vs. 15 mg					2.05 (1.15–3.65)	0.015		
**Hypertension**								
yes/ no	0.55 (0.32–0.97)	0.039	2.14 (1.03–4.46)	0.041				
**Diabetes mellitus**								
yes/ no	1.95 (1.12–3.39)	0.018						
**CHF**								
yes/ no								
**Prior ischemic stroke/TIA**								
yes/ no					2.39 (1.32–4.31)	0.004	1.84 (1.20–2.81)	0.005
**Vascular disease**								
yes/ no								
**Hepatic dysfunction**								
yes/ no			2.28 (1.10–4.74)	0.027				
**Oral antiplatelet use**								
yes/ no	3.45 (1.55–7.67)	0.002	2.51 (1.25–5.04)	0.009	4.61 (2.18-9.74)	<0.001		

Stepwise regression analysis was performed with a 5% significance level. Corresponding HR and P values were shown. CHF, congestive heart failure; CI, confidence interval; CrCl, creatinine clearance; HR, hazard ratio; TIA, transient ischemic attack.

Supplementary Table 4. Baseline patient characteristics

Characteristic	75- 79 years (N=2,083)	80- 84 years (N=1,571)	≥85 years (N=1,031)
Age, years	76.9 ± 1.4	81.8 ± 1.4	87.7 ± 2.8
Female sex	894 (42.92)	749 (47.68)	583 (56.55)
Body weight, kg	59.62 ± 11.76	56.32 ± 11.23	51.74 ± 11.07
Body weight, kg [n (%)]			
≤50	428 (20.55)	481 (30.62)	483 (46.85)
>50	1,519 (72.92)	971 (61.81)	462 (44.81)
Unknown	136 (6.53)	119 (7.57)	86 (8.34)
BMI, kg/m^2	23.86 ± 3.91	23.22 ± 4.20	22.10 ± 3.93
CrCl, ml/min	60.6 ± 18.7	51.5 ± 16.6	42.1 ± 14.1
CrCl, ml/min [n (%)]			
<50	529 (25.40)	714 (45.45)	718 (69.64)
≥50	1,403 (67.35)	724 (46.09)	221 (21.44)
Unknown	151 (7.25)	133 (8.47)	92 (8.92)
$CHADS_2$ score, mean ± SD	2.7 ± 1.1	2.9 ± 1.2	3.0 ± 1.2
Score, n (%)			
0	0 (0.00)	0 (0.00)	0 (0.00)
1	221 (10.61)	151 (9.61)	75 (7.27)
2	840 (40.33)	511 (32.53)	336 (32.59)
3	511 (24.53)	411 (26.16)	277 (26.87)
4	353 (16.95)	315 (20.05)	207 (20.08)
5	125 (6.00)	150 (9.55)	112 (10.86)
6	33 (1.58)	33 (2.10)	24 (2.33)
25th percentile	2	2	2
Median	2	3	3
75th percentile	3	4	4
CHA_2DS_2-VASc score	4.2 ± 1.2	4.4 ± 1.3	4.6 ± 1.3
Score			
0	0 (0.00)	0 (0.00)	0 (0.00)
1	0 (0.00)	0 (0.00)	0 (0.00)
2	118 (5.66)	79 (5.03)	31 (3.01)
3	531 (25.49)	307 (19.54)	170 (16.49)
4	679 (32.60)	465 (29.60)	323 (31.33)
5	438 (21.03)	390 (24.82)	249 (24.15)
6	235 (11.28)	226 (14.39)	159 (15.42)

7	70 (3.36)	89 (5.67)	80 (7.76)
8	10 (0.48)	15 (0.95)	19 (1.84)
9	2 (0.10)	0 (0.00)	0 (0.00)
25th percentile	3	4	4
Median	4	4	4
75th percentile	5	5	6
Modified HAS-BLED score*	1.8 ± 0.8	1.9 ± 0.9	2.0 ± 0.9
Score			
0	0 (0.00)	0 (0.00)	0 (0.00)
1	934 (44.84)	557 (35.46)	322 (31.23)
2	784 (37.64)	643 (40.93)	433 (42.00)
3	290 (13.92)	305 (19.41)	223 (21.63)
4	66 (3.17)	58 (3.69)	49 (4.75)
5	9 (0.43)	8 (0.51)	3 (0.29)
6	0 (0.00)	0 (0.00)	1 (0.10)
7	0 (0.00)	0 (0.00)	0 (0.00)
8	0 (0.00)	0 0.000	0 (0.00)
25th percentile	1	1	1
Median	2	2	2
75th percentile	2	2	3
Baseline comorbidities			
Hypertension	1,641 (78.78)	1,243 (79.12)	799 (77.50)
Diabetes mellitus	463 (22.23)	319 (20.31)	175 (16.97)
Prior ischemic stroke/TIA	482 (23.14)	480 (30.55)	343 (33.27)
Congestive heart failure	476 (22.85)	491 (31.25)	405 (39.28)
Hepatic dysfunction	99 (4.75)	83 (5.28)	46 (4.46)
Type of AF			
Paroxysmal	736 (35.33)	487 (31.00)	270 (26.19)
Persistent	710 (34.09)	581 (36.98)	411 (39.86)
Permanent	509 (24.44)	387 (24.63)	255 (24.73)
Other	7 (0.34)	3 (0.19)	2 (0.19)
Unknown	121 (5.81)	113 (7.19)	93 (9.02)

Data are presented as n (%) or mean ± standard deviation.

*Maximum score is 8 because of the exclusion of the factor "labile INR" from the HAS-BLED score.

AF, atrial fibrillation; BMI, body mass index; CrCl, creatinine clearance; INR, international normalized ratio; TIA, transient ischemic attack
